# Antibiotic‐induced reduction of abnormal lung shadow in pulmonary nodular lymphoid hyperplasia

**DOI:** 10.1002/rcr2.522

**Published:** 2020-02-05

**Authors:** Akari Tanino, Yukari Tsubata, Shunichi Hamaguchi, Akihisa Sutani, Mamiko Nagase, Takeshi Isobe

**Affiliations:** ^1^ Department of Internal Medicine, Division of Medical Oncology and Respiratory Medicine Shimane University Faculty of Medicine Izumo Japan; ^2^ Sutani Clinic Pulmonary Medicine Izumo Japan; ^3^ Department of Organ Pathology Shimane University Faculty of Medicine Izumo Japan

**Keywords:** Antibiotics, Actinomyces, lung, nodular lymphoid hyperplasia, pseudolymphoma

## Abstract

Pulmonary nodular lymphoid hyperplasia (PNLH) involves proliferative lymphatic tissues and is reportedly associated with inflammatory disease or autoimmune disorders. Herein, we describe a case of PNLH with difficult diagnosis because of antibiotics therapy‐induced reduction in the abnormal tumour shadow. An 86‐year‐old man was admitted for persistent cough and bloody sputum. Computed tomography (CT) revealed a mass in the right middle lobe, which got smaller on treatment with tosufloxacin for pneumonia. Unexpectedly, the tumour shadow remained one month later. Positron emission tomography depicted fluorodeoxyglucose uptake at the site. Although lung cancer was suspected, the mass was non‐diagnostic on transbronchial and CT‐guided biopsies. He was eventually diagnosed with PNLH on post‐surgical histological analysis of the lung mass. Neutrophil accumulation and bacterial lumps were present, indicating Actinomyces infection in the pulmonary alveolus, suggesting that PNLH was associated with pneumonia. Histopathological examination helped identify the aetiology of this rare case of PNLH.

## Graphical abstract

A case of pulmonary nodular lymphoid hyperplasia (PNLH) that was difficult to diagnosis because of an abnormal shadow that was reduced due to antibiotics use was described. Histopathological examination indicated a diagnosis of PNLH and the pathological evidence of bacteria suggested an infective aetiology for PNLH.

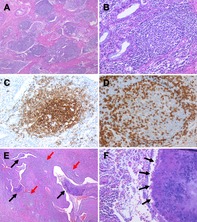

## Introduction

Pulmonary lymphoproliferative disorders (LPD) are characterized by nodal or diffuse infiltration of lymphoid cells into the lung parenchyma. LPD are also classified as reactive or neoplastic based on developmental processes. Pulmonary nodular lymphoid hyperplasia (PNLH) consists of nodules or localized lung infiltration by reactive lymphoid cells 1; it is a benign form of LPD with reactive changes. Although several cases of PNLH due to inflammation or combined with autoimmune disease have been reported, the developmental mechanisms involved in such cases are unclear. Herein, we report a case of PNLH that was evidently caused by infection.

## Case Report

**Figure 1:**
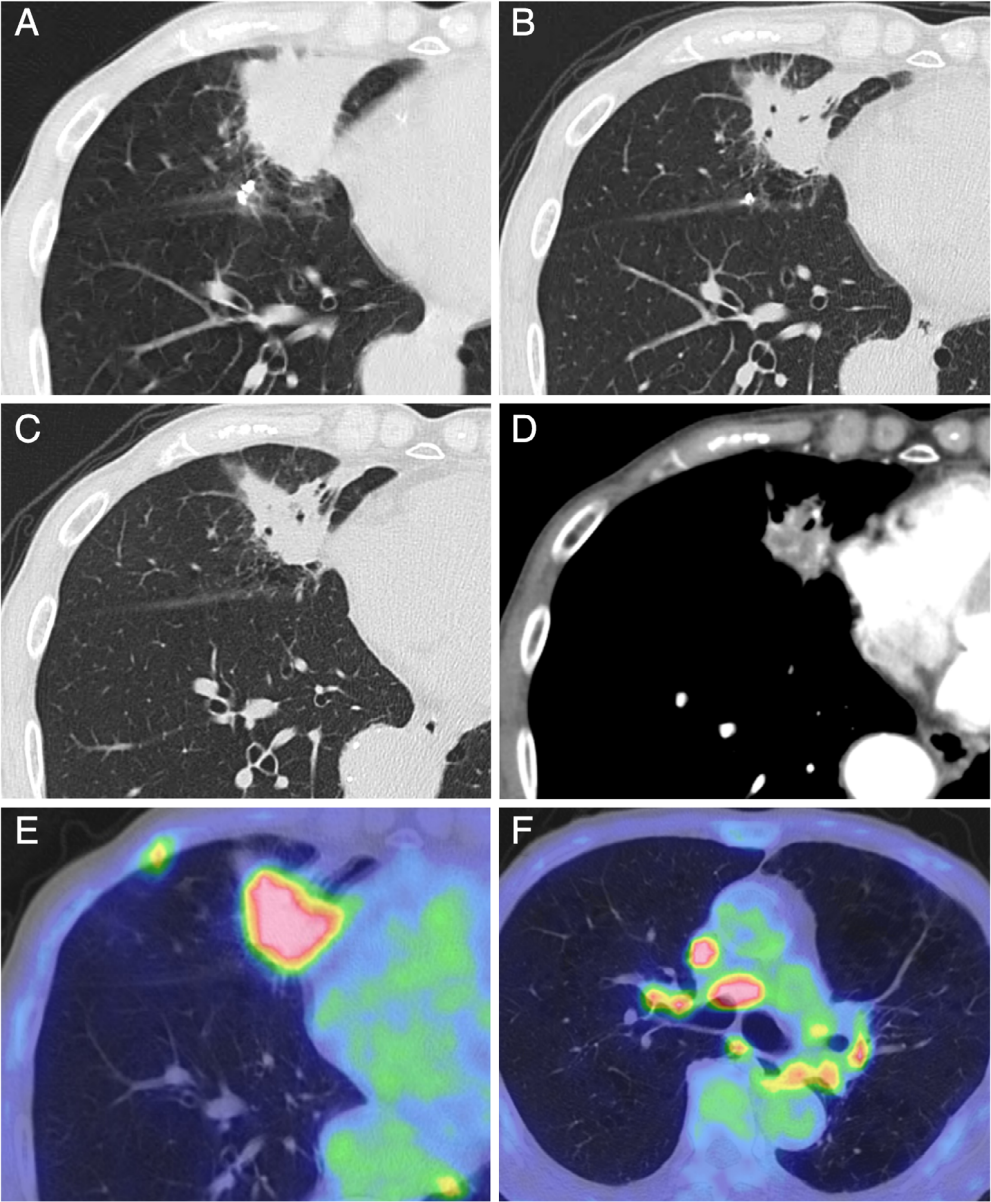
(A) Chest computed tomography (CT) scanning reveals a mass in the right middle lobe. (B) Tosufloxacin was administered for pneumonia, which shows reduction of the mass after one month. (C) CT scanning performed again after an additional one month unexpectedly shows the persistence of the tumour shadow. (D) An enhanced chest CT scan (mediastinal window setting). (E) Positron emission tomography depicts fluorodeoxyglucose uptake in the mass. (F) Fluorodeoxyglucose uptake is also observed in the lymph nodes.

An 86‐year‐old man presented with chief complaints of cough for the last three months and bloody sputum for the last one month. He had a 38 pack‐year smoking history. Chest computed tomography (CT) revealed a 48 × 42‐mm tumour shadow in the right middle lobe (Fig. 1A). He was administered tosufloxacin for nine days for suspected pneumonia. CT performed after one month depicted the tumour shadow had reduced in size to 43 × 35 mm after the treatment (Fig. 1B); therefore, tosufloxacin was changed to clarithromycin, and the treatment was continued for 14 days. Subsequent chest CT performed after additional one month showed persistence of the tumour shadow, with a size of 39 × 35 mm (Fig. 1C, and enhanced chest CT scan (mediastinal window setting) (Fig. 1D). The patient was admitted to our hospital for further examination because of suspected primary lung cancer.

**Figure 2:**
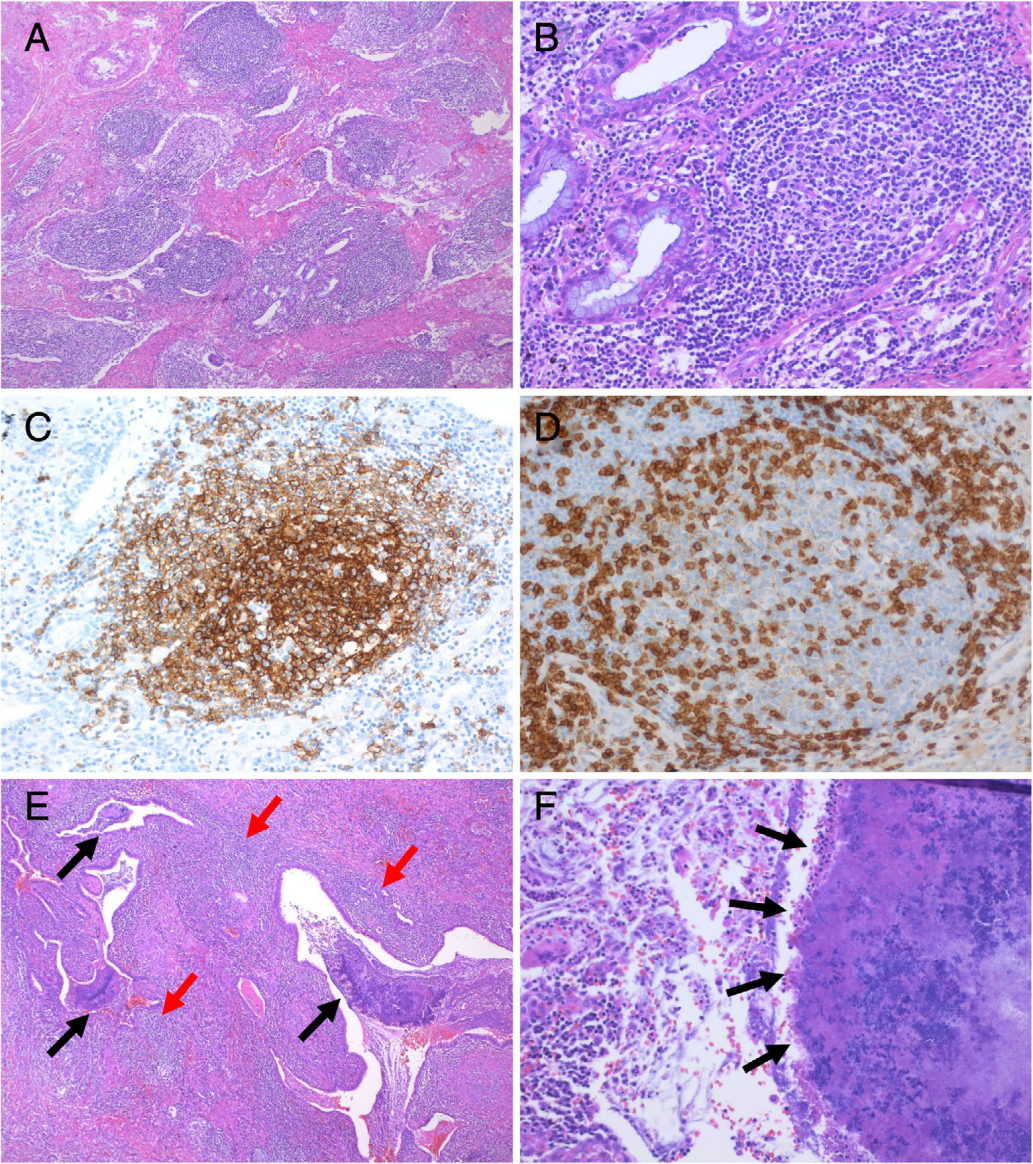
(A) Histological findings in a thoracoscopic lung biopsy specimen. Numerous lymphoid follicles, interfollicular fibrosis, and (B) benign lymphoid aggregates are present (haematoxylin and eosin staining). (C, D) Immunohistochemical staining shows reactive B and T cells. (C) The germinal centres show immunopositivity for CD20, a B cell marker, and (D) the interfollicular lymphocytes show immunostaining for CD3, a T cell marker. (E, F) Histopathological staining of the alveolar cavity and bronchus shows neutrophil aggregation (haematoxylin and eosin staining). Black arrows indicate bacterial masses suspected of being Actinomyces. Red arrows indicate the part of pulmonary nodular lymphoid hyperplasia (PNLH). The mass of Actinomyces (black arrows) was found mainly in the bronchus, and PNLH (red arrows) was mainly composed of lymphoid cells that diffusely infiltrated into the stroma. PNLH was present adjacent to Actinomyces (E: 20×, F: 200×).

Bilateral breath sounds were attenuated. There were slight elevations in carcinoembryonic antigen (7.2 ng/mL) and sialyl‐Lewis X (58.8 U/mL). Sputum examinations for acid‐fast bacteria and fungi were negative. No anti‐mycobacterium antibody was detected, but the mycoplasma antibody titre was increased to 640. Positron emission tomography revealed increased [^18^F]‐fluorodeoxyglucose uptake in the mass (maximum standardized uptake value: 7.8) (Fig. 1E). Fluorodeoxyglucose uptake was also observed in the right subclavian lymph node, right hilar lymph node, and right lower paratracheal lymph node (Fig. 1F). Bronchoscopy was performed for suspected primary lung cancer. Right B^5^b was rubbed and washed but the biopsy was subsequently cancelled because of bleeding. CT‐guided lung biopsy was performed. The mass was designated as class II on cytodiagnosis of the bronchoscopy samples, and class III on examinations of the CT‐guided lung biopsy samples. Therefore, a thoracoscopic lung biopsy was performed to facilitate a more definitive diagnosis. The gross lesion was accompanied by capillary enlargement, and it was considered more likely to be an inflammatory condition than a malignant tumour. Partial resection of the right middle lobe was performed, and there was no finding of malignant tumour on rapid pathological examination. Histologically, the mass showed numerous lymphoid follicles with interstitial fibrosis (Fig. 2A). The lymphocytes within the follicle had no heterotypic cells (Fig. 2B). Immunohistochemically, most cells of the germinal centres were CD20‐positive and bcl‐2‐negative B cells (Fig. 2C). CD3‐positive T cells were conspicuous around the marginal zone and the follicle, and exhibited a polyclonal pattern (Fig. 2D). These findings indicated that the lesions were reactive rather than neoplastic, and the case was diagnosed as PNLH. In the alveolar space and the bronchus, there were neutrophil clumping and bacterial masses suspected of being Actinomyces (Fig. 2E and 2F). PNLH was present adjacent to Actinomyces. The patient remains without PNLH recurrence after having undergone a partial resection of the lung.

## Discussion

PNLH is a benign disease involving polyclonal lymphoid proliferation, and it was previously referred to as pseudolymphoma 2. Histologically, PNLH comprises lymphoid follicles with germinal centres that have typical reactive features and large numbers of plasma cells between them. Interfollicular fibrosis is also commonly present. Immunohistochemical staining of a PNLH specimen typically reveals a reactive pattern of B and T lymphocytes 3. The majority of cases are detected as incidental lesions on radiological imaging conducted for other purposes. Some patients exhibit symptoms of shortness of breath, coughing, and pleuritic chest pain. The most common radiological pattern is solitary pulmonary nodule. Surgical resection is generally considered sufficient for treatment; the prognosis is usually good and recurrence is uncommon 4. A few cases associated with autoimmune diseases, such as Sjögren syndrome, have been reported 5.

In the present case, both histological and immunohistochemical characteristics were consistent with a diagnosis of PNLH. Neutrophil clumping and bacterial masses were evident in the alveolar space. We believe that pulmonary actinomycosis reactively triggered the formation of lymphoid follicles.

Although treatment response is slow and long‐term antibiotic administration (at least two months) is necessary, high doses of penicillin or tetracycline can effectively treat Actinomyces infection. Furthermore, reports indicate successful therapeutic efficacy with new‐generation fluoroquinolone. In this case, prolonged tosufloxacin regimen may have effectively treated Actinomyces infection, but it was prescribed only for nine days. Therefore, presumably, its therapeutic efficacy was not apparent at all sites of Actinomyces infection; only the acute inflammation surrounding the infection improved, with marginal reduction of the tumour shadow. Moreover, the hyperplastic mass itself remained unaffected by antibiotics. This made it difficult to differentially diagnose PNLH from an inflammatory condition.

There are two previously reported cases that exhibited focal clusters of neutrophils, which was identified as the cause of the inflammation in pathological examinations. One case involved a microabscess in association with bronchiectasis 4, and the other involved perilesional organized pneumonia 4. Both these reports and the present case suggest that inflammatory stimuli may be conducive to the development of prominent follicular lymphoid masses.

Both Actinomyces and PNLH were found in the biopsy specimen of the middle lobe tumour; therefore, fluorodeoxyglucose uptake may have been the result of either of the two. However, in this case, fluorodeoxyglucose uptake was also observed in lymph nodes. There are reports of cases in which lymph node enlargement has been observed with PNLH 4. Thus, although lymph node biopsy was not performed in this case, we consider the fluorodeoxyglucose uptake to be mainly due to the effect of PNLH.

PNLH is a rare disease whose aetiology remains poorly understood. Early surgical resection is important to differentially diagnose PNLH and malignant lesions. The current case is important because the pathological evidence of bacteria suggested an infective aetiology of PNLH. In cases of pneumonia with an abnormal shadow, which shows limited response to antibiotic therapy, a confirmatory biopsy and consequent histopathological examination must be conducted to exclude the presence of hyperplasia/cancer.

### Disclosure Statement

Appropriate written informed consent was obtained for publication of this case report and accompanying images.
